# Testicular Dysgenesis Syndrome and Long-Lasting Epigenetic Silencing of Mouse Sperm Genes Involved in the Reproductive System after Prenatal Exposure to DEHP

**DOI:** 10.1371/journal.pone.0170441

**Published:** 2017-01-13

**Authors:** Ludwig Stenz, Jessica Escoffier, Rita Rahban, Serge Nef, Ariane Paoloni-Giacobino

**Affiliations:** 1 Department of Genetic Medicine and Development, University of Geneva Faculty of Medicine, Geneva, Switzerland; 2 Swiss Centre for Applied Human Toxicology, Basel, Switzerland; Universite Clermont Auvergne, FRANCE

## Abstract

The endocrine disruptor bis(2-ethylhexyl) phthalate (DEHP) has been shown to exert adverse effects on the male animal reproductive system. However, its mode of action is unclear and a systematic analysis of its molecular targets is needed. In the present study, we investigated the effects of prenatal exposure to 300 mg/kg/day DEHP during a critical period for gonads differentiation to testes on male mice offspring reproductive parameters, including the genome-wide RNA expression and associated promoter methylation status in the sperm of the first filial generation. It was observed that adult male offspring displayed symptoms similar to the human testicular dysgenesis syndrome. A combination of sperm transcriptome and methylome data analysis allowed to detect a long-lasting DEHP-induced and robust promoter methylation-associated silencing of almost the entire cluster of the seminal vesicle secretory proteins and antigen genes, which are known to play a fundamental role in sperm physiology. It also resulted in the detection of a DEHP-induced promoter demethylation associated with an up-regulation of three genes apparently not relevant for sperm physiology and partially related to the immune system. As previously reported, DEHP induced an increase in mir-615 microRNA expression and a genome-wide decrease in microRNA promoter methylation. A functional analysis revealed DEHP-induced enrichments in down-regulated gene transcripts coding for peroxisome proliferator-activated receptors and tumor necrosis factor signaling pathways, and in up-regulated gene transcripts coding for calcium binding and numerous myosin proteins. All these enriched pathways and networks have been described to be associated in some way with the reproductive system. This study identifies a large new array of genes dysregulated by DEHP that may play a role in the complex system controlling the development of the male reproductive system.

## Introduction

The adverse impact of bis(2-ethylhexyl) phthalate (DEHP) on the animal reproductive system has been documented since the 1980s. In mice, DEHP was found to induce phenotypic alterations partially analogous to the human testicular dysgenesis syndrome (TDS) [1]. The increasing incidence of TDS was reported to be due to adverse environmental influences, but the exact mechanisms causing the disorder have still to be elucidated [2, 3]. At present, the disorder is thought to originate during fetal growth and to be associated with an alteration in testosterone production. Due to the impossibility to study the mechanisms that give rise to TDS in the human fetus, animals exposed *in utero* to phthalate are used as a mimicking model system [3].

In humans, evidence suggests that DEHP alters testosterone production and/or distribution, both essential for testes development. In a study using human testes cells in culture, DEHP 10^−5^ or 10^−4^ M or mono(2-ethylhexyl) phthalate (MEHP), the major DEHP metabolite, were found to inhibit testosterone production [4]. A large multicenter study showed a statistically significant association between blood levels of the sex hormone-binding globulin (SHBG) and urinary concentrations of MEHP [5]. The crystal structure of dihydrotestosterone (DHT) was found to bind to SHBG via a steroid binding pocket. Interestingly, fit docking simulations predicted that DEHP would bind with a higher affinity to the SHBG steroid pocket than DHT [6]. When occurring in a critical testis development period, competition between DEHP and DHT to bind to SHBG might induce a decrease in testosterone production, which may explain DEHP-induced TDS.

DNA methylation, histone modifications, and the expression of non-coding regulatory RNAs are well-known epigenetic mechanisms responsible for the diversification of various cell types during development and for the maintenance of diverse genomic expression profiles throughout life. “The primordial germ cells, embryo, and fetus are highly susceptible to epigenetic dysregulation by environmental chemicals, which can thereby exert multiple adverse effects” [7]. Previously, we identified promoter methylation changes in sperm DNA associated with decreased spermatogenesis in C57BL/6J susceptible mice prenatally exposed to DEHP [8].

DEHP exposure after complete testis development is also deleterious for reproductive functions. When testes and ovaries from 7 to 12-weeks-old human fetuses were exposed to 10^−4^ M MEHP during 72 h, an increased apoptosis was observed in male gonads. In addition, an increased expression of LXRα and its downstream genes involved in lipid and cholesterol synthesis was observed in both male and female gonads [9]. Treatment of Big Blue^®^ transgenic mice with DEHP for 4 weeks induced a 3-fold increase in genomic DNA mutation frequency [10]. Furthermore, a 96 h exposure to 10 μM MEHP of cultured chicken postnatal testis cells altered seminiferous tubule differentiation [11]. Similarly, a 30 min exposure to 1 μg/ml DEHP of mouse spermatozoa reduced their fertilization ability and the resultant embryo developmental potential [10]. These results strongly suggest that DEHP exposure has a direct negative impact on spermatozoa, independent of the alteration of testosterone production in testis.

The testis differentiation period seems to be the most critical time window to investigate the negative impact of DEHP on reproductive functions in males and to mimick human TDS, which occurs during fetal life and is associated with the abnormal development of testosterone-producing Leydig and supporting Sertoli cells. In mice embryos, somatic gonadal precursors composed of post-migrating primordial germ cells started the dynamic sexual differentiation to produce fetal testis with Sertoli, Leydig and pro-spermatogonial cells at embryonic day 10 (E10.5). This process is triggered by the “sex-determining region of Y chromosome” and involves the activation of testosterone-dependent genes [12, 13]. A previous investigation of TDS mechanisms showed that prenatal exposure to 500 mg/kg dibutyl phthalate during embryonic days 13 to 21 in Wister rats resulted in a significant reduction in plasma testosterone at day 25. A 90% reduction of testosterone levels was measured in testes at embryonic day 19, as well as the detection of Leydig cell hyperplasia, a dysgenic area, and the presence of immature Sertoli cells in testes [3].

We investigated the effects of DEHP administered to pregnant mice between days E9 and E19 (i.e. during the period that is considered as critical for testis differentiation) on the reproductive function *in vivo* and the genome-wide RNA expression and promoter methylation in the sperm of male offspring. We used also a new method that was able to quantify any type of RNA molecules at the genome-wide level, independent of their 5’ and 3’ modifications.

## Materials and Methods

### Mice mating and DEHP exposure

Animals were housed in plastic cages with food (RM3, SDS Dietex, France) and water provided ad libitum and maintained on a 12:12 light cycle. The zero filial generation (F0) consisted of 10 to 12-weeks-old male and female mice pairs of strain C57BL/6J (Charles River, l’Arbresle, France). Mice were mated in a cage during the night before selection and examined for the presence of a plug indicating copulation the following day, which was defined as embryonic day 1 (E1) by referring to future pups. F0 males were removed from the cage at E1. Prenatal exposure was performed by *per os* administration once daily from E9 to E19 to pregnant female mice restrained in one hand by grasping the scruff of the neck of 20 μl of either corn oil (Sigma, C8267) for controls (CTL) or 20 μl of 1.15 M DEHP (Fluka-Sigma, DEHP Selectophore, Catalog no. 80030) diluted in corn oil for exposed mice. The dose calculated for an estimated mouse weight of 30 g corresponded to 300 mg of DEHP per kg of mice per day (mg/kg*bw/day) (D300). The first filial generation of mice (F1) consisted of pups born at E21, weaned at postnatal day 21 (P21), and used for both computer-assisted sperm analyses (CASA) and RNA-seq experiments 100 days after birth (P100). Of note, a double dose of 20 μl of 2.3 M DEHP in corn oil, corresponding to 600 mg/kg*bw/day (D600) of DEHP, was additionally used in experiments recording litter sizes.

### Anogenital distance measurements

The anogenital distance (AGD) was expressed in centimeters and corresponded to the distance separating both anal and genital orifices. It was measured by two investigators in F1 live males using a graduated ruler.

### CASA and sampling

Five CTL male and five D300 male F1 offspring of the treated females were sacrificed 100 days after birth by C0_2_ inhalation followed by cervical dislocation. Left and right testes were dissected from the animal bodies and weighed. One was fixed in Bouin solution and the other in 4% paraformaldehyde phosphate-buffered saline solution. The left and right cauda epididymides of each animal were incised with a scalpel in 1 ml M2 medium and maintained at 37°C during 10 min. Diluted sperm suspensions were loaded into a 37°C pre-warmed chamber slide of 100 μm depth. The slide was observed in a phase contrast microscope. Sperm fertility parameters were measured using the CEROS II CASA system (Hamilton Thorne Research, Beverly, MA) and data were analyzed using the CASAnova software. Five microscopy fields were analyzed in each experiment and amounted to at least 700 spermatozoa per sample. The CASA recorded the following parameters: 1) the sperm concentration expressed in millions of spermatozoa per ml; 2) the percentage of motile spermatozoa; 3) the curvilinear velocity in μm/s; and 4) the amplitude of lateral head displacement in μm/s.

### Testes histology

Ten different testes (five per condition) were fixed in Bouin, embedded in paraffin, sliced with a microtome, stained with hematoxylin-eosin, and analyzed under a microscope at four different magnifications. A total of 483 seminiferous tubule diameters and 483 corresponding lumen diameters were measured in μm at 5X magnifications (48 ± 12 tubules measured per sample). Classification of seminiferous tubules according to the spermatid presence in lumens were performed at 10X magnifications for five CTL F1 and five D300 F1 samples, resulting in a total of 141 tubules segregated as “negative”, “closed”, “positive” and “full”. “Negative” tubules contained lumen with an apparent absence of elongated spermatids. “Closed” tubules corresponded to tubules without lumen. “Positive” tubules contained lumen with small numbers of spermatid. “Full” tubules contained lumen massively colonized by flagella of elongating spermatids.

### RNA extraction from spermatozoa

For each mouse, 900 μl of sperm suspension not used for CASA were carefully obtained by pipette in order to avoid scratched left and right cauda epididymis tissues and centrifuged at 17,000 g during 30 sec before replacement of the liquid phase with 1 ml of the TRIzol^®^ Reagent (Ambion, Austin, TX). The purity of spermatozoa used to extract RNA was assessed before the procedure by visualizing sperm under a contrast phase microscope during the CASA process and after RNA sequencing with read length distributions corresponding to the profile of mature sperm RNA signatures as previously determined [14]. RNA extraction was performed according to the manufacturer’s recommendations and included the addition of 10 μg RNase-free glycogen as carrier (Invitrogen, UltraPure^™^ Glycogen, Carlsbad, CA) during RNA precipitation in isopropanol. The final elution was in 20 μl water (Bioconcept, Water for Molecular Biology DNA/DNAse/RNAse free) and samples were frozen in dry ice and conserved at -80°C in labelled Eppendorf tubes.

### RNA-derived libraries construction

In general, messenger RNAs contain 3’-poly-A tails and a 5’-cap, but most mature microRNAs differ with a 5'-monophosphate extremity and a free 3’-hydroxyl group resulting from their maturation by Dicer cleavage. RNA samples were sent to Fasteris SA (Geneva, Switzerland) in dry ice. RNA quality was verified with the Bioanalyzer 2100 Expert or the Caliper GX-LST LabChip^®^ systems (Caliper Life Sciences, Massachusetts, USA). First, ribosomal RNAs were depleted using the Ribo-Zero rRNA removal kit (Illumina Inc, San Diego, CA; MRZH116 or MRZG12324). Second, rRNA-depleted RNA was fragmented to sizes in the range of 60–200 nt using a fragmentation reagent (Ambion; AM8740). Third, RNA molecules were treated to be compatible with adapter ligation following a protocol developed by Fasteris SA: i) RNA molecules naturally carrying various pyrophosphate bonds and 5'-cap structures were converted in RNA carrying a 5'-monophosphate group using Cap-Clip^™^ acid pyrophosphatase treatment; ii) 5’-monophosphate-RNAs were treated with phosphatase-producing RNAs without any phosphate group in 5' and 3'; iii) the phosphate group free RNA molecules were treated with T4 polynucleotide kinase-producing 5’-monophosphate RNA molecules. Even if deriving from mRNA and microRNA, the resulting molecules should carry a 5'-monophosphate extremity and a free 3’-hydroxyl group. Libraries were then prepared using the TruSeq small RNA kit (Illumina Inc.) following the manufacturer’s instructions and involving the RNA 3’ adapter (RA3) 5’-TGGAATTCTCGGGTGCCAAGG and RNA 5’ adapter (RA5) 5’-GUUCAGAGUUCUACAGUCCGACGAUC.

### Sequencing and base calling

Libraries were sequenced on a HiSeq 2500 (Illumina Inc.). Base calling was performed using HiSeq Control Software 2.2.58, RTA 1.18.64.0 and CASAVA-1.8.2. The data discussed in this publication have been deposited in the NCBI Gene Expression Omnibus [15] and are accessible through GEO series accession number GSE86837 (http://www.ncbi.nlm.nih.gov/geo/query/acc.cgi?acc=GSE86837).

### RNA-seq analysis

Fastq files were uploaded and the analysis was performed in-house in a Linux environment (Ubuntu 14.04 LTS) containing the installed bioinformatic tools required. Quality control of the sequencing process was performed with fastqc ensuring >Q28 in the quality control report for all reads. RA3 was clipped from fastq files and the minimum reads size was settled at 18 pb using the command line: $ fastx_clipper -a TGGAATTCTCGGGTGCCAAGG -l 18 -i /data/PATH/original.fastq -o /data/PATH/C57_CTL_F1_1.fastq. The analysis was performed using TopHat and Cuffdiff [16]. Reads were mapped for each sample using TopHat to the mm10 mouse genome with the following command line: $ tophat -p 16 -G /data/PATH/genes.gtf—library-type fr-secondstrand -o /data/PATH /C57_CTL_F1_1 /data/PATH/genome /data/PATH/C57_CTL_F1_1.fastq. SAM files were indexed and sorted: $ samtools sort accepted_hits.bam C57.CTL.F1.1.sorted. $ samtools index C57.CTL.F1.1.sorted.bam. Quantification was performed with the following command line: $ cuffdiff -o /data/PATH/diff_out -b /data/PATH/genome.fa -p 16 -L CTL,D300 -u /data/genome/mm10_new/Mus_musculus/UCSC/mm10/Annotation/Genes/genes.gtf /data/PATH/C57.CTL.F1.1.sorted.bam,/data/PATH/C57.CTL.F1.2.sorted.bam,/data/PATH/C57.CTL.F1.3.sorted.bam,/data/PATH/C57.CTL.F1.4.sorted.bam,/data/PATH/C57.CTL.F1.5.sorted.bam, /data/PATH/C57.D300.F1.1.sorted.bam,/data/PATH/C57.D300.F1.2.sorted.bam,/data/PATH/C57.D300.F1.3.sorted.bam,/data/PATH/C57.D300.F1.4.sorted.bam,/data/PATH/C57.D300.F1.5.sorted.bam. The reads log table was built to control and resume processes performed from fastq to bam files (Table 1).

**Table 1:** Read log sheet of RNA-seq experiments.

Sample	Reads produced	Clipped reads >18 bp	Clipped reads lost ratio	Length of clipped reads	Reads mapping mm10	Mapped reads lost ratio
C57_D300_F1_1	62,370,005	48,072,079	0.23	18–50	41,581,189	0.14
C57_D300_F1_2	61,289,415	50,599,210	0.17	18–50	44,084,120	0.13
C57_D300_F1_3	64,705,541	52,738,583	0.18	18–50	45,347,087	0.14
C57_D300_F1_4	73,423,174	58,363,679	0.21	18–50	48,878,978	0.16
C57_D300_F1_5	60,720,003	52,309,334	0.14	18–50	45,704,724	0.13
C57_CTL_F1_1	43,268,415	36,994,852	0.14	18–50	30,080,705	0.19
C57_CTL_F1_2	68,710,604	59,640,671	0.13	18–50	50,884,558	0.15
C57_CTL_F1_3	65,719,341	57,936,661	0.12	18–50	48,286,489	0.17
C57_CTL_F1_4	58,952,279	52,332,091	0.11	18–50	43,032,461	0.18
C57_CTL_F1_5	58,665,529	51,691,382	0.12	18–50	39,121,345	0.24

### Pathway analysis

Statistically significant dysregulated genes defined by the CuffDiff output table were clustered into two groups using a K-means clustering approach to segregate correctly down-regulated genes in D300 in cluster 1, and up-regulated genes in D300 in cluster 2. Separate enrichment analysis of pathways for up- and down-regulated genes were performed to identify enrichments and networks of interacting genes based on “a database of known and predicted protein-protein interactions”, including “direct (physical) and indirect (functional) associations” identified by the STRING database and using the most stringent parameters [17]. The analysis was performed online (http://string-db.org/) with the two lists of official gene symbols and selecting the “*Mus musculus*” organism. The minimum required interaction score was set at the highest confidence level (0.9) and only networks involving more than two genes were taken in account. Otherwise, dashed lines display interactions that do not resist the highest confidence level. Separate enrichment analysis of pathways for up- and down-regulated genes was preferred. According to a previous report, this approach is more powerful than analyzing all of the differentially expressed genes together and results in the identification of “more pathways that are really pertinent to phenotyping differences” [18].

### Combined promoter methylation and RNA expression analysis

The RNA-seq database was merged by gene symbol in the R free software environment (Project for Statistical Computing; https://www.r-project.org/) with our previously published and open access methyl-CpG binding (MBD)-seq database (http://www.ncbi.nlm.nih.gov/geo/query/acc.cgi?acc=GSE67159) used under similar conditions for strain C57BL/6J with CTL and DEHP300 subjects [8]. Regression analysis was performed using the locally weighted scatterplot smoothing (LOWESS) method to estimate the trend between RNA expression and promoter methylation. Gene selection for combined MBD-seq and RNA-seq analyses was done in two steps. First, statistically significant differentially-expressed genes in CuffDiff output associated with the lowest and the highest fold changes were selected as the top-up and top-down targets. Second, the nine most closely expressed genes for both targets were then fished using the Jensen-Shannon distance estimate. For statistical differences in the measurement of fragments per kilobase of transcripts per million fragments mapped (FPKM) between both conditions, i.e. p values derived from the non-parametric Mann-Whitney-Wilcoxon test performed among five CTL and five D300 mice, the means and 95% confidence interval were calculated, independent of the nature of the measurement distribution.

### Statistical analysis

A non-parametric Mann-Whitney-Wilcoxon test was performed when measurements of reproductive parameters did not follow a normal distribution; Student's *t*-tests were performed for parameters following a normal distribution. Pearson's chi-squared test (χ2) was performed in R using the “chisq.test” function on seminiferous tubule segregation results obtained for both F1 CTL and F1 D300 conditions. Basic statistical analysis of the validity of the differential analyses from quintuplicate sperm-extracted RNA samples between both CTL and D300 conditions was performed using CummeRbund version 2.14.0. Data quality was estimated both at the post-sequencing step and before differential analysis. Differential analysis of RNA was performed at the genome level without a priori. The global densities of FPKM measurements across genes were verified to be similar between both conditions and across the 10 samples at the genome scale (data not shown). Statistical significant differences in FPKM measurements between CTL and D300 conditions were taken in account after Benjamini-Hochberg correction for multiple testing implemented within CuffDiff.

### Ethical statement

This study was approved by the Ethics Committee for Animal Experimentation of the University of Geneva Medical School and by the Geneva cantonal Veterinarian Office (permit reference: G61/3918) under the license number GE/9/15. Mice were purchased from Charles River (France) and maintained at the animal core facility of the University of Geneva Medical School under conventional accommodation. All animal manipulation was monitored using the Python-based Relational Animal Tracking system. Animals were housed and cared for according to the ethical guidelines of the Canton of Geneva Ministry of Health.

## Results

### Testis dysgenesis and affected reproductive parameters after prenatal exposure to DEHP

**Fig 1:**
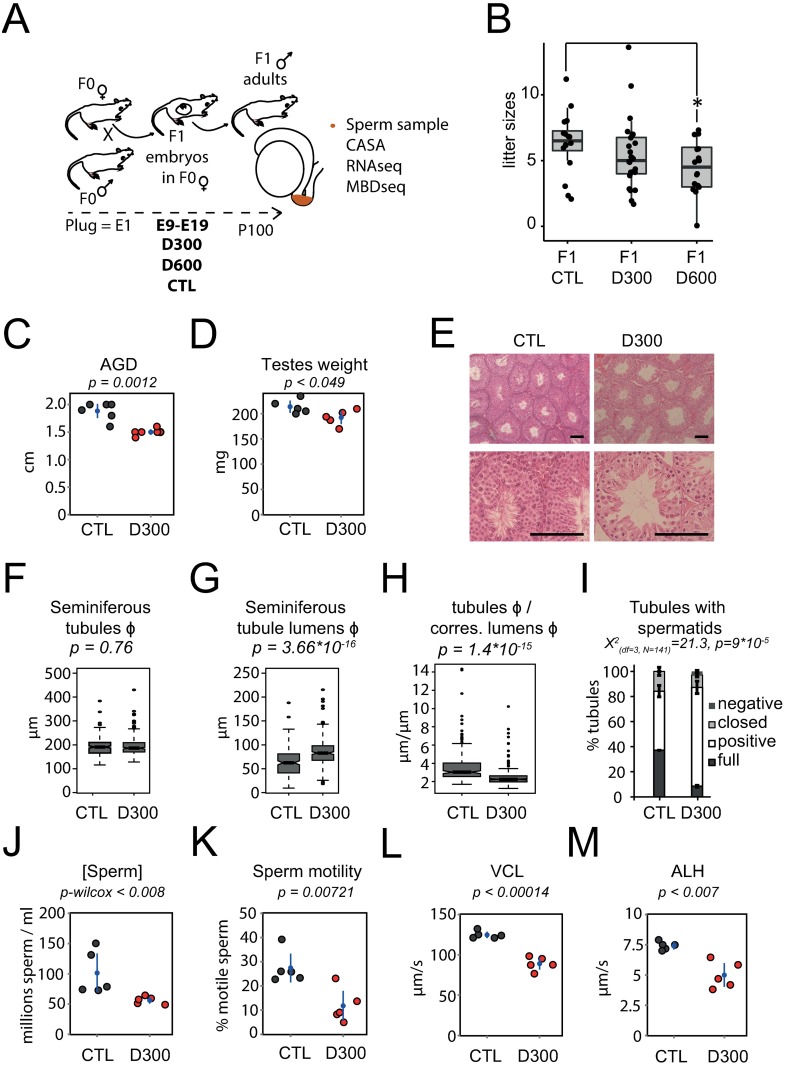
Testis dysgenesis and decreased reproductive parameters after prenatal exposure to DEHP. **(A)** Experimental design. Prenatal exposure occurred at embryonic days 9 to 19 (E9-E19) with corn oil (CTL), 300 mg/kg/day DEHP (D300), or 600 mg/kg/day DEHP (D600). **(B)** Litter sizes among groups by conditions. **(C, D, E, F, G, H, I, J, K, L, M)** Reproductive parameters measured in F1 CTL and D300 males. **(C)** Anogenital distance (cm) in D300 compared with CTL mice. **(D)** Testicular weight or weights of both testes (mg) in D300 compared with CTL mice. **(E)** Bouin-fixed, microtome-sliced and hematoxylin-eosin-strained testis observed at 10X (up) and 40X (down) magnifications of CTL (left) and D300 mice (right). A black scale bar in the lower right corner of each image corresponds to 100 μm. **(F**, **G**, **H)** Box plot measurements in μm obtained from measurements performed in 483 seminiferous tubules (264 in D300 and 219 in CTL mice). Measurements are: **(F)** seminiferous tubule diameters; **(G)** lumen diameters; and **(H)** tubule diameters over corresponding lumen diameters. **(I)** Stacked bar charts of seminiferous tubules segregated as “negative”, “closed”, “positive” and “full” presenting significant differences between both CTL (n = 70) and D300 (n = 71) conditions (Chi-square p-value = 9*10^−5^). **(J)** Sperm concentration measured in millions of spermatozoa per ml in D300 compared with CTL mice. **(K)** Sperm motility measured as percentages of motile sperm cells in D300 compared with CTL mice. **(L)** Sperm curvilinear velocity measured in μm per second in D300 compared with CTL mice. **(M)** Sperm amplitude of lateral head displacement measured in μm per second in D300 compared with CTL mice. **(C**, **D**, **K**, **L**, **M)** Indicated p-values derived from parametric two-tailed Student’s t-tests performed between CTL and D300 mice. **(J)** A non-parametric Mann-Whitney-Wilcoxon test was performed (Wilcox p-value). **(C, D, J, K, L, M)** Dots represent measurements obtained from individual F1 mice: CTL (gray dots) and D300 (red dots). Points are spread on the x-axis to avoid overlap. Means and 95% confidence intervals are shown as blue dots and blue bars, respectively, and were calculated independent of the nature of the distribution of the measurements. AGD: Anogenital distance. VCL: Curvilinear velocity. ALH: amplitude of lateral head displacement. Df: degree of freedom. N: total number of tubules segregated.

A possible impact of prenatal DEHP exposure on the litter sizes of offspring was tested. The litter sizes of F1-treated mothers (F1 D300) decreased compared with control mothers (F1 CTL), but this effect was significant only for 600 mg/kg/day (F1 D600), which was the highest dose tested (t-test; p = 0.03) (Fig 1B).

The impact of fetal DEHP exposure on male reproductive parameters was investigated in F1 males born from mice receiving either 20 μl corn oil vehicle (CTL) or 300 mg/kg/day DEHP diluted in corn oil (D300) *per os* each day from E9 to E19. CASA was performed on sperm extracted from the cauda epididymis (Fig 1A) dissected at 100 days in C57BL/6J males (P100). Compared with CTL subjects, all reproductive parameters measured were significantly decreased in the F1 offspring of mice prenatally exposed to DEHP. AGD and testes weight were significantly reduced in the D300 group compared to CTL mice, (1.9 cm ± 0.16 in CTL vs. 1.5 cm ± 0.06 cm in D300 [p = 1,2*10^−3^] and 214 mg ± 14 in CTL vs. 193 mg ± 15 in D300 [p = 0.049], respectively) (Fig 1C & 1D). Histological observation of the testes showed a reduction of the germinal tissue thickness inside the seminiferous tubules in D300 compared to CTL mice (Fig 1E). This apparent reduction was quantified by measuring the diameters of the tubules, but was not significant (192 μm ± 38in CTL vs. 193 μm ± 39 in D300; p = 0.76) (Fig 1F). Of note, the diameters of the lumens significantly increased in D300 compared with CTL mice (64 μm ± 28 in CTL vs. 86 μm ± 29 in D300; p = 3.66*10^−16^) (Fig 1G). The ratio of seminiferous tubules diameters over corresponding lumen diameters was reduced under D300 conditions (3.6 μm ± 1.9 in CTL vs. 2.5 μm ± 0.97 in D300; p = 1.4*10^−15^) (Fig 1H). Although the rate of seminiferous tubules with spermatids did not differ between CTL and D300 mice, seminiferous tubules with lumens massively colonized by flagella of elongating spermatids (“full”) were decreased in D300 compared with CTL mice and associated with a Chi-square-based significant difference of tubular segregation results (Fig 1I).

In the D300 group, the sperm concentration measured with CASA was almost half of that observed in the CTL mice (101 million per ml ± 56 in CTL vs. 56 ± 6 millions per ml in D300; Wilcox p-value = 7,9*10^−3^). The minimal CTL value (73 millions) remained higher than the maximal D300 value (64 millions) (Fig 1J). All motility-related parameters were reduced in D300 compared to CTL mice as follows. Motility: 27.4% ± 6.8 motile spermatozoa in CTL vs. 11.8% ±7 in D300; p = 7.21*10^−3^); curvilinear velocity: 125 ± 5 μm/s in CTL vs. 85 ± 8 μm/s in D300; p = 1.4*10^−4^); and amplitude of lateral head displacement: 7.4 μm/s ± 0.3 in CTL vs. 5 μm/s ± 1 in D300; p<0.007), (Fig 1K, 1L & 1M).

Overall, the prenatal exposure of C57BL/6J mice to 300 mg/kg/day of DEHP resulted in decreases in AGD, sperm concentration and motility and in seminiferous tubules’ germinal thickness, as well as in the proportion of “full” tubule, i.e. a phenotype consistent with a TDS.

### Sperm RNA expression changes after prenatal exposure to DEHP

**Fig 2:**
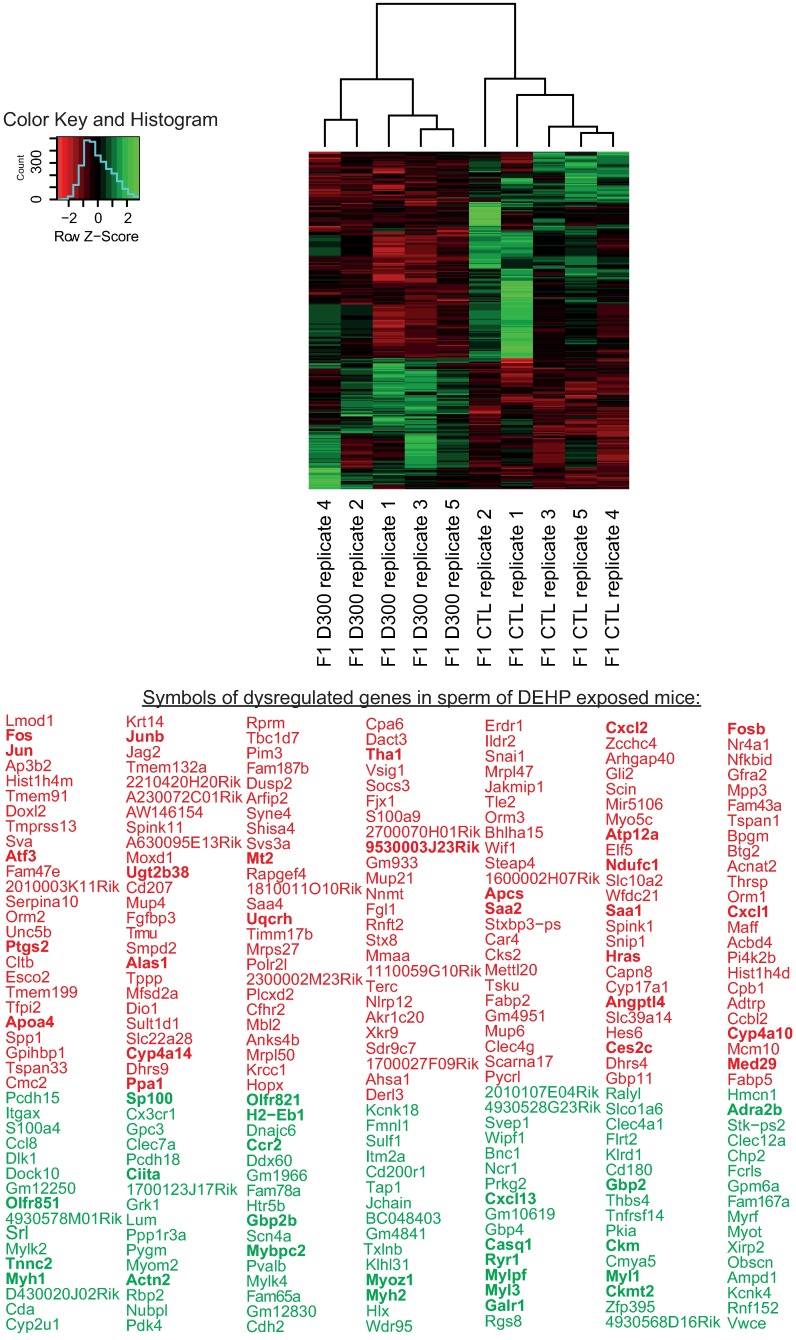
Sperm RNA expression changes after prenatal exposure to DEHP. Clustering and heat map analysis performed on the 283 genes that were significantly differentially present or expressed in sperm of controls (CTL) and DEHP exposed mice (D300). Data correspond to 10 RNA-seq experiments analyzed with TopHat-Cuffdiff pipeline [16]. Measurements were obtained in the five D300 individual samples clustering together on the left and five CTL individual samples clustering on the right of the heat map. The 173 genes down-regulated in D300 mice are presented under their official gene symbols in red; the 110 genes up-regulated in D300 mice are presented under their official gene symbols in green. The genes identified in a network are presented in bold.

We postulated that the defects observed in both the concentration and the motility of spermatozoa in D300 mice may be caused by alteration in gene expression involved in sperm survival and motility. To test this hypothesis, RNAs extracted from spermatozoa sampled from the cauda epididymidis were quantified with the modified RNA-seq process in quintuplicates in both CTL and D300 mice. Results showed that 283 genes were significantly differentially present or expressed. One hundred and seventy-three (61%) were down-regulated, whereas 110 (39%) were up-regulated in D300 compared to CTL mice (Fig 2). These genes were enriched in several pathways discussed later. Note that some genes measured previously by reverse transcription quantitative polymerase chain reaction (RT-qPCR) were used as controls (S1 Fig).

### Pathways in which mRNA expression is up- or down-regulated after prenatal exposure to DEHP

The genome-wide RNA quantification study was followed by pathway analysis to identify putative pathways dysregulated in D300 vs. CTL mice. Up- and down- regulated genes were separately submitted to online functional analysis using STRING [17], resulting in both the characterization of interacting networks of dysregulated genes and significant enrichments in functional pathways.

**Fig 3:**
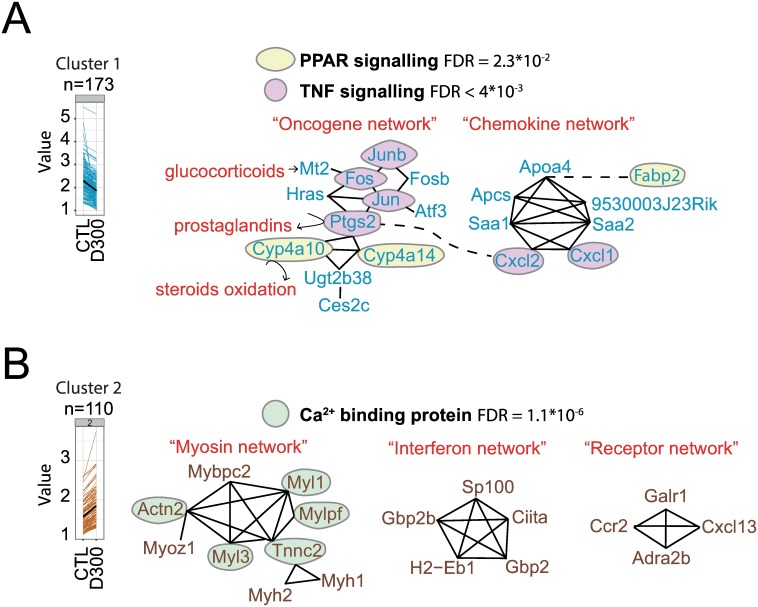
Dysregulated pathways in sperm after prenatal exposure to DEHP. **(A)** Members of cluster 1 (shown as blue line and blue gene symbols) correspond to 173 genes significantly down-regulated in D300 compared to CTL mice. Cluster 1 is significantly enriched for the TNF (FDR = 4*10^−3^) (pink circles) and PPAR signaling pathways (FDR = 2*10^−2^) (yellow circles). Two networks of interacting genes can be identified that share members of both the TNF and PPAR signaling pathways. First, the oncogene network displays 12 genes among which Fos, Jun, Jumb and Ptgs2 belong to the TNF pathway and Cyp4a10 and Cyp4a14 to the PPAR pathway. Second, the chemokine network displays seven genes among which Cxcl2 and Cxcl1 belongs to the TNF pathway. Hormones and prostaglandins (in red) interact with members of the oncogene network (black arrows). Glucocorticoid hormone-induced Mt2 expression and hormone-like prostaglandins are synthesized by prostaglandin-endoperoxide synthase 2 (Ptgs2) and steroid oxidation is catalyzed by both cytochromes Cyp4a10 and Cyp4a14 (see results section). **(B)** Cluster 2 (in brown) is composed of 110 genes significantly up-regulated in D300 mice and is highly significantly enriched in calcium ion binding proteins (FDR = 1.1*10^−6^). Three networks of genes were identified: the myosin network (nine members), the interferon network (five members), and the receptor network (four members). Calcium ion binding proteins belonging to the myosin network are Actn2, Myl1, Mylpf, Tnnc2 and Myl3 (green circles). The interferon network involves the tumor suppressor Sp100 and both antimicrobial peptides related to Gbp2b and Gbp2 and a HLA class 2 member, all sharing a common immune response. The receptor network involves coding receptors of three genes: the galanin receptor (Galr1), the C-C chemokine receptor type 2 (Ccr2), and the alpha-2B adrenergic receptor (Adra2b), although the last chemokine (C-X-C motif) ligand 13 (Cxcl13) is not a receptor.

In the 173 sperm down-regulated genes, significant enrichments of both the peroxisome proliferator-activated receptor (PPAR) and tumor necrosis factor (TNF) signaling pathways were observed (Fig 3A). In addition, the functional analysis revealed the enrichment of two interacting gene networks, i.e. the oncogene network and the chemokine network. Interestingly, both networks shared members of both signaling pathways. As DEHP interferes with mammalian hormones, the possible involvement of genes associated with hormone metabolism was also investigated. Mt2, Cyp4a10 and Ptgs2 genes in the oncogene network have been previously involved in the metabolism of glucocorticoids, steroids, and prostaglandins, respectively (Fig 3A).

In the 110 sperm up-regulated genes, significant enrichments of calcium-binding proteins (false discovery rate (FDR) = 1.1*10^−6^)) were observed, as well as in three networks of interacting genes, i.e. the myosin network, interferon network, and receptor network, apparently disconnected from each other (Fig 3B). The myosin network contains calcium-binding proteins associated with myosin interacting genes and consists of numerous genes encoding myosin constituents, such as the myosin light polypeptides (Myl1, Myl3), heavy polypeptides (Myh1, Myh2), light chain (Mylpf), light polypeptide kinase (Mylk2, Mylk4, not in the network), myosin-binding protein (Mybcpc2), and obscurin (Obscn, not in the network), the last putative gene involved in the assembly of myosin (Fig 3B). Genes involved in the metabolism of hormones were not clearly identifiable in the networks.

### Associations of RNA expression and promoter methylation changes after prenatal exposure to DEHP

In mice prenatally exposed to DEHP, experimental evidence suggests an alteration of spermatogenesis. The latter involves the dynamic expression and epigenetic changes of genes across various developmental stages ranging from spermatogonium to mature elongated spermatids, as well as interactions between different cell subtypes. Genes whose RNA expression was highly dysregulated by prenatal exposure to DEHP associated with promoter methylation changes were analyzed in mature sperm and then confronted to normal spermatogenesis-related expression profiles to investigate if these targets were associated with a particular spermatogenesis step.

Dysregulation of sperm RNA expression in D300 mice may be caused by the methylation of CpG in promoters, which are able to block transcriptional activity by rendering DNA inaccessible to transcription factors. In order to test if prenatal exposure to DEHP could mediate a long-lasting promoter methylation gene silencing in the sperm, RNA-seq was merged with accessible MBD-seq data (http://www.ncbi.nlm.nih.gov/geo/query/acc.cgi?acc=GSE67159) that consists of sequencing methylated DNA after its capture by affinity using the MBD domain. Both datasets were obtained under similar conditions and compared CTL and D300 C57BL/6J mice [8].

**Fig 4:**
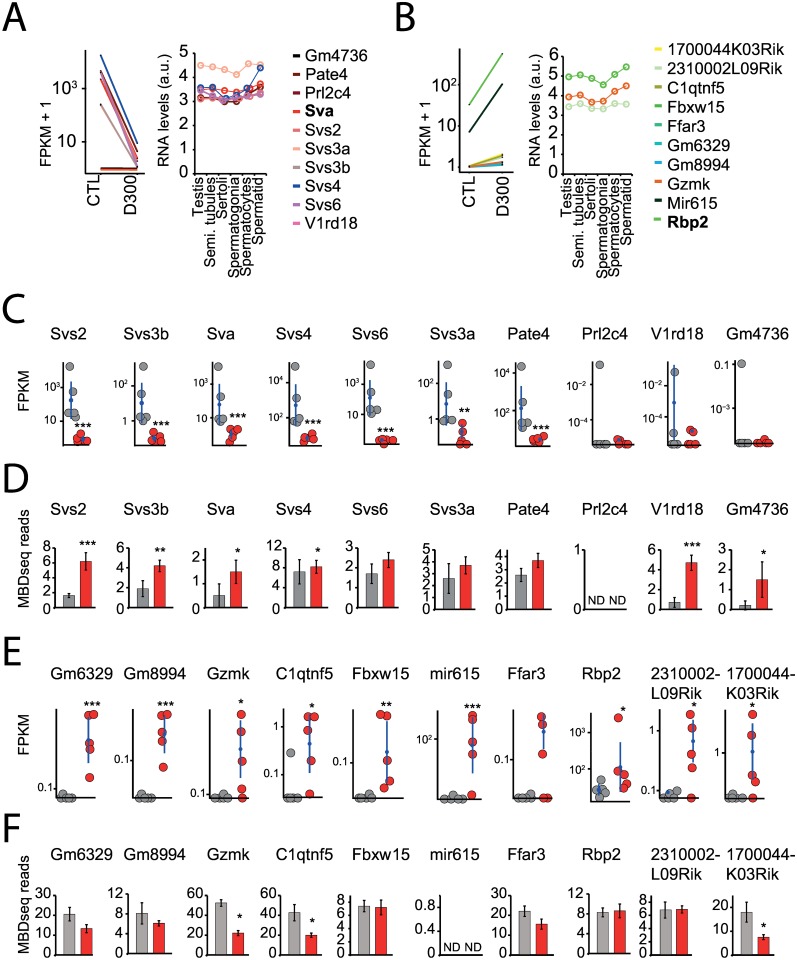
Sperm-selected genes with RNA expression and promoter methylation changes after prenatal exposure to DEHP. **(A,B,C,D,E,F)** Promoter methylation analysis in the most dysregulated genes in terms of fold changes between CTL and D300 conditions combined with assessment of the transcriptional levels across spermatogenesis-related cells and tissues. **(A, B)** When compared with CTL conditions, most down- and up-regulated genes in the D300 group fished by Jensen-Shannon distance estimates performed with the topmost down and significant *Sva* and the topmost up and significant Rbp2 genes (shown in bold). Expression data from Chalmel F et al. (2007) recorded in C57BL/6J mice sampled testis and sperm cell subpopulations for the genes identified in the present study. **(C, E)** Individual FPKM values recorded for the dysregulated genes in D300 (red dots) compared with CTL samples (gray dots). Means and 95% confidence intervals in both CTL and D300 conditions are indicated as blue dots and bars. **(D, F)** Mean promoter methylation values in CTL (gray bars) and D300 (red bars) recorded in C57BL/6J sperm MBD-seq experiments (Prados J et al. [2015]) expressed in MBD-seq reads. Errors bars represent standard deviations. *** p< 0.008. ** p< 0.01. * p< 0.05. ND: no data.

Decreased RNA expression with increased promoter methylation was observed in the entire dataset for both CTL and D300 mice (data not shown). We analyzed the most dramatically dysregulated genes in terms of fold changes in RNA expression using Jensen-Shannon distance estimates. Genes whose FPKM status was exactly equal to zero in one condition (CTL or D300) were removed for mathematical reasons (Fig 4).

Among the 10 top down-regulated genes in the sperm of DEHP exposed mice, six of the eight known members of genes encoding seminal vesicle secretory proteins or antigens were identified: *Svs2*, *Svs3a*, *Svs3b*, *Svs4*, *Svs6*, *Pate4*, and *Sva* (Fig 4C). In four (*Svs2*, *Svs3b*, *Svs4*, *Sva*), a statistically significant decrease in mRNA expression associated with a statistically significant increase in promoter methylation in DEHP exposed mice was observed (Fig 4C & 4D). These genes are highly expressed in spermatogenesis-related tissues and sperm cells, in particular, the *Svs4* expression level increases with sperm cell maturation [19] (Fig 4A). *Svs2* was highly expressed according to the lacZ reporter (Svs2^tm1.1(KOMP)Vlcg^) in seminal vesicle, prostate, epididymidis and ductus deferens (http://www.informatics.jax.org/assay/MGI:5771180) [20].

Nine of the 10 top up-regulated genes identified by Jensen-Shannon distance estimates (*Gm6329*, *Gm8994*, *Gzmk*, *C1qntf5*, *Fbxw15*, *mir615*, *Rbp2*, *231002L09Rik* and *1700044K03Rik*) reached statistical significance (Fig 4E). Three (*Gzmk*, *Ctqtnf5* and *1700044K03Rik*) resulted in statistically significantly increased mRNA expression associated with decreased promoter methylation in the sperm of DEHP exposed mice (Fig 4E & 4F). *Gzmk*, *Rbp2* and *230002L09Rik* are transcripts in spermatogenesis-related tissues and sperm cells (Fig 4B), but there are no data concerning the tissue-specific mRNA expression in the remaining 10 top up-regulated genes [19]. It is noteworthy that among these 10 top up-regulated genes, one microRNA (mir615) was detected by Jensen-Shannon distance estimates. Promoter methylation of mir-615 was absent in the MBD-seq database.

### Expression and methylation changes in gene subtypes after prenatal exposure to DEHP

**Fig 5:**
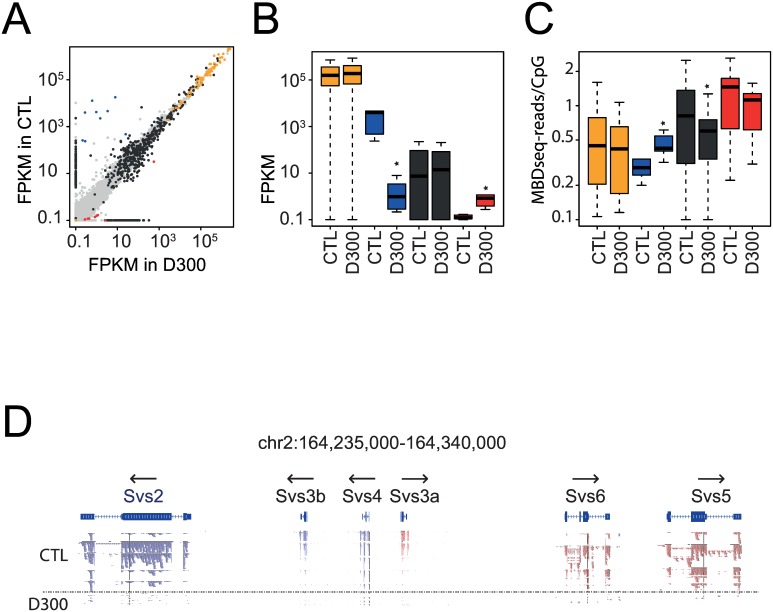
RNA expression and promoter methylation changes in genes coding for RNA subtypes after prenatal exposure to DEHP. **(A, B, C)** Analysis of transcript levels and promoter methylation across the RNA subtypes. Orange dots and bars represent sixty-seven detected C/D box *Snord*s. Blue dots and bars represent nine seminal vesicle secretory genes (*Sva*, *Svs1*, *Svs2*, *Svs3a*, *Svs3b*, *Svs4*, *Svs5*, *Svs6*, *and Pate4)*. Black dots and bars represent nine hundred and eighty-six microRNAs. Red dots and bars represent nine upregulated genes in the sperm of DEHP exposed mice (*Rbp2*, *mir615*, *C1qtnf5*, *Gzmk*, *Gm8994*, *Gm6329*, *Fbxw15*, *231002L09Rik*, and *1700044K03Rik)*. **(A)** Scatterplot of transcript levels in FPKM with the CTL group on the vertical axis and the D300 group on the horizontal axis. **(B)** Boxplot of FPKM expression across RNA subtypes and under experimental conditions. **(C)** Boxplot of methylation in MBD-seq reads per CpG in 2.2 kb probed promoter regions across RNA subtypes and conditions. Stars represent significant differences between CTL and D300 groups (results are shown in Table 2). **(D)** Reads mapped to the cluster of *Svs* genes in chromosome 2 across the 10 samples. Five CTL samples up and five D300 down from the horizontal dashed line. Reads in blue and red differ according to their orientation; genes orientations are shown with arrows.

The profile of expression and promoter methylation across different type of RNAs was investigated to characterize to what extent both RNA-seq and MBD-seq data were in agreement, to test if the functionally related genes were altered at the gene group level, and to investigate the global behavior of the microRNAs. Genes were grouped in the following subtypes. The first group consisted of 67 detected C/D box small nucleolar RNAs (*Snord)*. The second group (*Svs*) was composed of the nine seminal vesicle secretory genes (*Sva*, *Svs1*, *Svs2*, *Svs3a*, *Svs3b*, *Svs4*, *Svs5*, *Svs6*, and *Pate4*). All microRNAs were grouped together in a “mir” group (n = 986). The last group (“up”) consisted of the nine top genes detected as up-regulated in the sperm of DEHP exposed mice (*Rbp2*, *mir615*, *C1qtnf5*, *Gzmk*, *Gm8994*, *Gm6329*, *Fbxw15*, *231002L09Rik* and *1700044K03Rik*). Results showed that both *Snord* and *Svs* were the two most highly expressed RNA subtypes in the sperm of CTL mice associated with less methylation in promoters, whereas microRNAs and the “up” gene groups were the two less highly expressed RNA subtypes in the sperm of CTL mice associated with more methylation in promoters (Fig 5A, 5B & 5C). These results are in accordance with the model of promoter methylation-mediated repression of gene expression. At the gene group levels, statistically significant differences were observed in the sperm of DEHP exposed mice compared to CTLs regarding expression in the *Svs* (decrease: Wilcox p-value = 4.1*10^−5^) and “up” (increase: Wilcox p-value = 2.8*10^−3^) groups, whereas significant promoter methylation differences were detected in the *Svs* (increased: p = 0.04) and microRNA (decrease: p< 2*10^−16^) groups (Table 2).

**Table 2:** Statistics of transcripts and promoter methylation levels in sperm RNA subtypes.

RNA subtypes	mean CTL FPKM	mean D300 FPKM	p-value	mean MBD-seq reads per CpG in CTL	mean MBD-seq reads per CpG in D300	p-value
“Snord”	414133	415015	0.76	0.76	0.46	0.23
“**Svs**”	5191	4.5	**0.00004**	0.37	0.48	**0.04**
“Mir”	9639	8312	0.086	0.89	0.56	<2.2*10–16
“Up”	3.66	63.3	**0.003**	0.65	0.41	0.34
“others”	207	245	0.35	0.39	0.34	0.29

Bold: significant differences between CTL and D300 conditions.

Despite the highly significant decrease observed in microRNA promoter methylation, the expression of microRNA analysis did not show any statistical difference. High variability and numerous cases of microRNAs showing 0 FPKM in one condition was observed. The difference in expression of the six *Svs* genes clustered in the mouse chromosome 2 was verified at the reads mapping level. Results showed visible difference of reads coverage between D300 and CTL mice, as well as a correct orientation of the mapped reads according to the gene orientations (Fig 5D).

## Discussion

We showed that prenatal exposure to DEHP in C57BL/6J mice resulted in statistically significant lower litter sizes at the highest dose tested, compatible with decreased embryo survival. F1 males were bred up to mature adulthood at P100 and five F1 D300 were compared to five CTL subjects. Results demonstrated a reduced AGD, lighter testes, severe decreased sperm quality, atrophy of the germinal tissue thickness, and a lower amount of seminiferous tubules with lumens full of elongated spermatids (Fig 1). These observations converge to an acquired hypogonadism at adulthood and a phthalate-induced phenotype resembling human TDS. The reduction of fertility in males prenatally exposed to DEHP remains to be further analyzed by detailed recordings of litter sizes, plug productions and pregnancy efficiency in the second filial generation (F2). F2 mice should be obtained by crossing F1 D300 and F1 CTL males with new females, thus producing comparable F2 litters. In addition, testosterone supplementation experiments are strongly needed. In the absence of both results, the impact on fertility and interferences with testosterone remains speculative in the present study.

The observed phenotype is in accordance with previous work reporting on prenatal exposure to phthalates inducing TDS in rodents [1]. Even if highly speculative, we believe that the observed DEHP-induced TDS may be caused by the impaired distribution of androgen produced by Leydig cells during the critical period for embryonic testis differentiation with a potential effect on reproductive performance. Recently, an *in silico* approach revealed that the DEHP molecule may compete with DHT and/or testosterone for SHBG [6]. AGD is a biomarker of androgen exposure during the fetal testis development period in rodents as well as in humans [21], whereas sperm concentration and motility are the most significant predictor of human fertility [22].

Epigenetic effects of endocrine disruptors have been extensively investigated using targeted approaches [23–26]. We have previously shown evidence for DEHP-induced differentially methylated promoters in sperm [8]. To identify functional DMRs associated with an altered transcription or RNA presence, we tested a new hypothesis that DEHP may exert its deleterious effects on spermatogenesis by also affecting paternally-transmitted sperm RNA.

Sperm cell cytoplasm phagocytosis by Sertoli cells during spermatogenesis and haploid DNA packaging by protamines in mature spermatozoa triggered the misunderstanding that spermatozoa were devoid of RNA and should escape transcription. The discovery that paternal RNA remains present inside spermatozoa and is passed over to the oocyte has shifted this paradigm [27]. A nuclease-hypersensitive and transcriptionally-competent conformation of chromatin in mouse sperm was discovered to be associated with transcription factors [28]. Ribosomal RNA (rRNA) was first considered to be absent from spermatozoa [29]. However, it was then identified as present, but cleaved [30], and recently estimated to represent 70% of total sperm RNA with the potential to predict *in vitro* fertilization prognosis [31]. In a study by Drosha and Dicer performed in the sperm of conditional knock-out mice lacking mature microRNAs, the injection of wild-type, sperm-derived RNA or small RNA into oocytes during *in vitro* fertilization rescued the pathological phenotype [32]. The sperm-borne microRNA “miR-34c” was found to be important for the first cell division via modulation of Bcl-2 expression [33]. All these observations showed that paternal RNA may contribute to embryo development.

RNA-seq analysis segregated correctly the samples in the two tested conditions (Fig 2). Pathway analysis resulted in the characterization of down-regulated PPAR and TNF signaling pathways combined with up-regulated myosin coding genes associated with calcium binding proteins in the D300 condition (Fig 3A & 3B). Indeed, in the current scientific literature, these pathways were described to be involved in spermatozoa physiology and, for some, as a mediator of DEHP toxicity [34–47].

First, docking simulation performed with DEHP or its metabolite MEHP with both PPAR alpha and gamma receptor subtypes showed that DEHP and MEHP could bind to PPAR alpha and gamma subtypes, with a lower binding affinity for DEHP [34]. Moreover, various studies on the role of PPAR signaling in spermatozoa suggested its involvement in promoting spermatozoa motility across different organisms. PPAR transcripts were significantly higher in high motile semen samples of rams [35]. Pig spermatozoa motility was increased by the PPAR-ɣ ligand PGJ2 and decreased by the specific GW9662 PPAR-ɣ antagonist [36]. Activation of PPAR-ɣ was required for spermatozoa motility in humans [37]. However, experiments performed with PPAR alpha-null (-/-) and wild-type (+/+) male *Sv*/129 mice revealed that PPAR alpha-independent pathways contributed at least partially to both renal and testicular DEHP toxicity [38]. Second, a negative impact of TNF-alpha was observed on spermatozoa motility and TNF-blocking therapies are currently under study for the treatment of infertility [39, 40]. MEHP induced a stabilization of p53 expression in spermatocytes and an enhancement of a TNF-related apoptosis-inducing ligand (TRAIL) pathway [41]. In a TNF-alpha knockout mouse model, testicular testosterone levels were decreased and associated with delayed spermatogenesis, reduced testis weight, and hypospermia [42], whereas others reported a down-regulation by TNF-alpha of the Müllerian inhibiting substance in Sertoli cells [43]. Third, calcium ion is considered as a key regulator of human sperm function, notably of spermatozoa motility [44], whereas myosins, belonging to a superfamily of ATP-dependent motor proteins regulated by calcium signals, are involved in different steps of the spermatogenesis process, as recently reviewed [45]. In brief, myosin participates in the assembly and positioning of the spindle during karyokinesis in meiosis, binds to chromosomes and segregates them by attachment to the microtubules. It then, participates in the acrosomal formation and spermatid individualization [45]. An impact of DEHP on calcium signaling and/or myosins was observed in studies involving other cell types than spermatozoa and performed in biological contexts other than reproductive toxicology. DEHP suppressed the calcium signaling of human nicotinic acetylcholine receptors in human neuroblastoma [46] and induced a decrease in myotube formation in C2C12 cells, associated with reduced MyHC, MyoD and myogenin levels [47].

Combining MBD-seq to RNA-seq performed under similar conditions, a putative functional epigenetic alteration in seminal vesicle secretory (*Svs)* genes and antigen was discovered in the present study (Figs 4 & 5; Table 2). Genes coding *Svs*2, *Svs*3b, *Sva* and *Svs*4, which are functionally related to sperm physiology, were statistically significantly down-regulated and associated with statistically significant promoter methylation increases in D300 compared to CTL conditions (Fig 4C & 4D), thus compatible with a long-lasting silencing able to be observed months after the last exposure to the endocrine disrupter. No associations between *Svs* genes and DEHP toxicity were found in previous studies, whereas *Svs* genes were reported to play fundamental roles in spermatozoa physiology.

Both semenogelin-1 and -2, encoded by *Svs2* and *Svs3ab* genes in mice are the major protein constituents of seminal fluid. They represent 20% to 40% of its proteins and are responsible for the spontaneous coagulation of semen after ejaculation by the formation of a semi-solid gelatinous mass entrapping immotile spermatozoa. The prostate specific antigen (*Pate*4 or *Svs*7) rapidly cleaves both semenogelin-1 and -2 and results in semen liquefaction, the initiation of sperm motility, and the production of a peptide presenting antibacterial activity [48]. Furthermore, *Svs*2 (semenogelin-1) attached to ganglioside GM1 on the plasma membrane of the sperm head maintain sterols in place and prevent sperm capacitation in a wrong location or at a wrong time [49]. Interestingly, *Svs*2 knockout male mice were sub-fertile; their sperm did not form plugs and did not survive to artificial insemination [50]. Seminal vesicle secretory protein IV (*Svs*4) protects spermatozoa against reactive oxygen species and oxidative stress-induced apoptosis [51]. Both *Svs*4 and *Svs*6 expression are up-regulated by testosterone. Their transcriptional levels decreased in castrated male mice, but they were restored after administration of testosterone [51, 52]. Finally, the seminal vesicle antigen prevents spontaneous acrosome reaction by targeting membrane sphingomyelin and plasma membrane Ca(^2+^)-ATPase activity [53].

To validate our hypothesis of DEHP-induced de-silencing events, i.e. activation of genes that are normally kept silenced by promoter methylation in the sperm, we screened in the 10 top up-regulated genes increased mRNA expression levels associated with decreased promoter methylation levels in D300 compared to CTL conditions. We identified Gzmk, encoding Granzyme K serine protease usually detected in cytoplasmic granules in both cytotoxic T-lymphocytes and natural killer cells, C1qtnf5 (bicistronic with Mfrp) involved in cell adhesion, as well as non-coding RNA 1700044K03Rik of an unknown function, whereas mir615 (overlapping Hoxc5) RNA expression was significantly dysregulated, but lacked promoter methylation data (Fig 4E & 4F). These genes were not reported as involved in sperm physiology, except for mir615, which is putatively involved in gonadal differentiation [54]. A novel granzyme involved in spermatogenesis was identified in mouse spermatocytes and spermatids [55].

To conclude, prenatal exposure to DEHP induced a TDS-like syndrome in F1 adult male mice, associated with a putative epigenetic silencing of *Svs* genes in sperm. The results of this study may be strain-specific and cannot be extrapolated as such to other mouse strains. The next steps of the present large scale analytic study would be to determine to what extent our discoveries are transposable to humans. For instance, it would be interesting to investigate if SEMG1 (*Svs*2 in mice), the gene encoding semenogelin 1, is up-methylated and silenced in association with the symptom of decreased sperm motility in infertile patients. Obvious further research would be to identify biological sperm signatures of human infertility that could be used as biomarkers by mining in the reservoir of the genes found in our study to be affected by prenatal exposure to DEHP. Finally, all the data collected here should allow to conduct additional fundamental studies aimed at understanding the complex pattern of genes controlling testis development and normal sperm physiology.

## Supporting Information

S1 Fig Expression levels assessed by RT-qPCR and RNA-seq on selected targets. (A) *piwil4*, (B) *piwil2*, (C) *igf2r*, (D) *rabl2* and (E) *star* expression levels measured previously by RT-qPCR (Prados et al, 2015) and in the present RNA-seq experiments from sperm samples in the C57BL/6J mouse strain comparing controls (CTL) to prenatal exposure to DEHP (D300). RT-qPCR data are expressed in arbitrary units. RNA-seq data are expressed FPKM. Expression differences between both CTL and D300 conditions were concordant between both RT-qPCR and RNA-seq approaches, except for the *star* gene, significantly over-expressed in the D300 condition according to RT-qPCR, but not in RNA-seq. (F) Sashimi plots generated from RNA-seq data showed a putative unannotated microRNA signature close to the binding site of the reverse primer used in RT-qPCR to measure *star*. As oligo-dT annealing to the poly-A tails of mature mRNA was used only in RT-qPCR and not in our RNA-seq, the discrepancy between RT-qPCR and RNA-seq measures of *star* expression levels may be explained by post-transcriptional regulation of *star*. Further experiments are needed to confirm the presence of this putative unannotated microRNA. AU: arbitrary units; FPKM: fragments per kilobase of transcript per million mapped fragments; RT-qPCR: reverse transcription quantitative polymerase chain reaction.
(TIF)
