# Supplementary figures and images for: Testicular Dysgenesis Syndrome and Long-Lasting Epigenetic Silencing of Mouse Sperm Genes Involved in the Reproductive System after Prenatal Exposure to DEHP
Source: PLoS One. 2017 Jan 13;12(1):e0170441. doi: 10.1371/journal.pone.0170441 (PMC5234833; doi:10.1371/journal.pone.0170441)

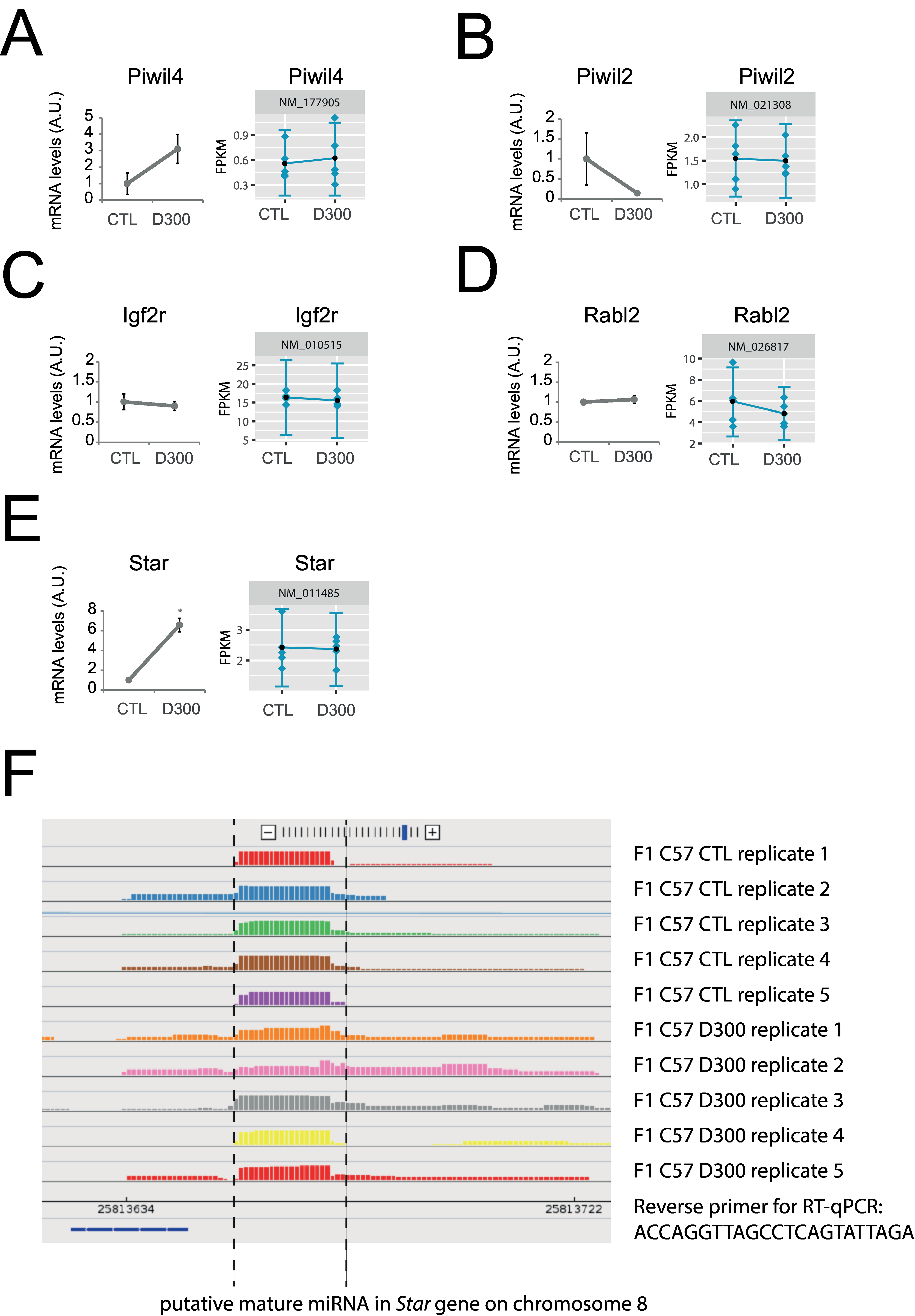

Supplement: S1 Fig — (A) piwil4, (B) piwil2, (C) igf2r, (D) rabl2 and (E) star expression levels measured previously by RT-qPCR (Prados et al, 2015) and in the present RNA-seq experiments from sperm samples in the C57BL/6J mouse strain comparing controls (CTL) to prenatal exposure to DEHP (D300). RT-qPCR data are expressed in arbitrary units. RNA-seq data are expressed FPKM. Expression differences between both CTL and D300 conditions were concordant between both RT-qPCR and RNA-seq approaches, except for the star gene, significantly over-expressed in the D300 condition according to RT-qPCR, but not in RNA-seq. (F) Sashimi plots generated from RNA-seq data showed a putative unannotated microRNA signature close to the binding site of the reverse primer used in RT-qPCR to measure star. As oligo-dT annealing to the poly-A tails of mature mRNA was used only in RT-qPCR and not in our RNA-seq, the discrepancy between RT-qPCR and RNA-seq measures of star expression levels may be explained by post-transcriptional regulation of star. Further experiments are needed to confirm the presence of this putative unannotated microRNA. AU: arbitrary units; FPKM: fragments per kilobase of transcript per million mapped fragments; RT-qPCR: reverse transcription quantitative polymerase chain reaction. (TIF) [file pone.0170441.s001.tif]
